# One size does not fit all: Assuming the same normal body temperature for everyone is not justified

**DOI:** 10.1371/journal.pone.0245257

**Published:** 2021-02-03

**Authors:** Adele Diamond, Carolyn T. Lye, Deepali Prasad, David Abbott

**Affiliations:** 1 Developmental Cognitive Neuroscience Program, Department of Psychiatry, University of British Columbia, Vancouver, British Columbia, Canada; 2 Sentinel Secondary, West Vancouver, British Columbia, Canada; 3 Crofton House, Vancouver, British Columbia, Canada; University of Lübeck, GERMANY

## Abstract

Despite the increasing personalization of medicine, surprisingly ~37.0°C (98.6°F) continues as the estimate of normal temperature. We investigated between-subject and within-subject thermal variability, whether a significant percentage of individuals have a low mean oral temperature, and whether these differ by sex, age, time of day, ethnicity, body mass index (BMI), or menstrual phase. Oral temperature was measured by Life Brand^®^ Fast-Read Digital Oral Thermometers and sampled 14 times over two weeks, seven morning and seven evening readings. The volunteer sample consisted of 96 adults (42 men, 54 women; 27 couples, 42 singletons), ages 18–67 years. We found sizeable individual differences in body temperature and that the normal temperature of many individuals is considerably lower than 37.0°C (98.6°F). Mean temperatures ranged from 35.2°C (95.4°F) to 37.4°C (99.3°F). The mean temperature across all participants was 36.1°C (97.0°F)—lower than most studies have reported, consistent with recent evidence of temperature declining over decades. 77% had mean temperatures at least 0.55°C (1°F) lower than 37.0°C (98.6°F). Mean temperature did not differ by age, but women had higher temperatures than men, even within a couple with room temperature and warmth of clothing equated. Although oral temperature varied widely across individuals, it showed marked stability within individuals over days. Variability of temperature over days did not differ by sex, but was larger among younger adults. Using 37.0°C (98.6°F) as the assumed normal temperature for everyone can result in healthcare professionals failing to detect a serious fever in individuals with a low normal temperature or obtaining false negatives for those individuals when using temperature to screen for COVID-19, mistaking their elevated temperature as normal. Some have called for lowering the estimate of normal temperature slightly (e.g., 0.2°C [0.36°F]). That still seems an overly high estimate. More important, using any standardized “normal” temperature will lead to errors for many people. Individual differences are simply too great. Personalizing body temperature is needed. Temperature could be measured at yearly doctor visits, as blood pressure is now. That would be simple to implement. Since our results show marked thermal stability within an individual, sampling temperature only once yearly could provide an accurate indication of a person’s normal temperature at that time of day. Such records over time would also provide a more accurate understanding of how temperature changes over the lifespan.

## Introduction

In these days of increasingly personalized medicine, a surprising relic is the continued assumption that 37.0°C (98.6°F) is a rough approximation of each person’s normal temperature. What if the normal temperature of many is over a degree less than that and some people’s normal temperature is as low as 35°C (95°F) or lower? That matters because serious fevers in such persons can go unidentified by medical professionals who see their elevated temperature as close to 37.0°. It also matters because checking people’s temperatures as a way to screen for COVID-19 will yield false negatives for people with low normal temperatures because their elevated temperatures are accepted as in the normal range by screeners.

We investigated between-subject and within-subject thermal variability, whether a significant percentage have low mean oral temperatures, and whether these differ by sex, age, time of day, ethnicity, body mass index (BMI), or menstrual phase. As far as we know, this is the first study to investigate (1) thermal stability and variability across days and weeks, (2) overnight temperature changes (from bedtime to waking) in a sizeable sample over multiple nights, and to compare (3) temperatures not only of unrelated men and women but also within couples, where room temperature and warmth of clothing were equated. Our main hypotheses were:

(I) (a) Sizeable inter-individual differences in body temperature exist and (b) the average temperature of many individuals is at least one degree fahrenheit (0.55°C) below 37.0°C (98.6°F). Even Wunderlich in his 1868 magnum opus [1] reported finding that “normal” temperature fell along a range, though that point has been largely ignored in clinical medicine [2]. Most studies over the past 50 years, especially those assessing oral temperature, have found a lower mean temperature than did Wunderlich. Many studies [e.g., 3] have found the difference to be as large as 0.55°C (1°F), i.e. mean temperature of ≤36.5°C (97.6°F), though a number have not [4] (36.57°), 5 (36.8°).
(II) A given individual’s temperature is fairly stable. We were primarily interested in inter-day reliability rather than diurnal fluctuation. We could no other investigation of the stability of a person’s temperature over days, weeks, or months.

Our secondary hypotheses were:

(III) Women have higher temperatures than men (as important studies have found, e.g. [5, 6]).
(IV) Evening temperatures are higher than morning temperatures (based on consistent reports of diurnal fluctuations in temperature with temperature being lowest in the morning [7, 8]).
(V) Women tend to be warmer in the evening and cool down by morning while men tend to be cooler in the evening and warm up by morning—predicted based on personal observations.
(VI) Younger adults have higher temperatures than older adults (given the much-replicated finding that temperature decreases with aging, e.g. [2–6, 9]).

It was not our intent to estimate population values. A far larger sample would have been needed for that. The intent was to see if we would find enough variability among individuals and enough people with mean temperatures ≤36.5°C (97.6°F) to support the recommendation that we not assume everyone’s normal temperature is ~37.0°C (~98.6°F), but instead routinely take a person’s temperature at each doctor visit, much as we do now with blood pressure. This would be simple to implement and could be life-saving for those whose normal body temperature is quite a bit lower than 37.0°C (98.6°F).

## Materials and methods

### Participants

Our sample consisted of 96 adults (42 men, 54 females; no one self-identified as neither or other), ranging in age from 18–67 years (mean = 30 years). Persons who were feeling unwell at the time or recently, were pregnant, had a chronic illness, or were taking any medication that could depress one’s temperature were excluded. Ethnicities of participants were 55% European origin, 16% East Asian, 16% South Asian, 8% Middle Eastern, and 5% mixed. No participant smoked. Participants were recruited via posters and social media, gave their written informed consent, and were paid $10 to thank them for their participation. No participant refused to participate since potential participants contacted us to volunteer. The study received human subjects approval from the University of British Columbia and Vancouver Coastal Health.

### Materials and procedure

Life Brand^®^ Fast Read Digital Oral Thermometers were used. They are accurate to 0.1°iover the range of 34.0° to 42.0°. To take their temperature, participants turned on the thermometer, placed it under their tongue towards the back of their mouth, closed their lips, and waited until the thermometer beeped.

Participants were to take seven pairs of temperature readings in the evening right before going to sleep and first thing the following morning over a two-week period (yielding a total of 1,333 temperature readings). Thus all temperature readings were taken in participants’ own homes in the greater Vancouver, BC area. Participants were instructed not to eat, drink, or engage in strenuous physical exercise during the 30 minutes prior to taking their temperature and not to take their temperature for this study when feeling unwell. We also collected data on factors that might affect body temperature including weight, height, age, ethnicity, and menstrual phase (when a woman’s last period began and ended and when she expected her next period). about the length of their menstrual cycle and dates of their last and next (estimated) periods.

We calculated BMI from height and weight (weight divided by height squared [kilograms/meters^2^). We estimated midluteal phase menstrual phase by counting back 6–8 days from the anticipated onset of the next menstruation, and early follicular phase by counting forward 3–5 days after the onset of menstruation. We did not ask about use of oral contraceptives unfortunately. All data were collected between January and May in Vancouver, BC, where the mean outdoor temperature during this period was 7°C (44°F) and ranged from -8°C (18°F) to 16°C (61°F).

Couples participating were asked to be in the same room and wear similarly heavy or light clothing at the time of the temperature readings and for ≥30 minutes beforehand to try expose their bodies to the same ambient environment with similar protection against heat or cold. Our sample included 27 couples and 42 singles. All couples were heterosexual.

We provided each participant with a thermometer and a temperature collection form for recording temperature readings and their dates and times. Importantly, participants were unaware of our hypotheses. Participants gave a $10 deposit for the thermometer, which was returned when they handed in their collection form and thermometer.

## Results

Repeated measures analysis of covariance (ANCOVA) was used for all analyses, except when comparing variances. To compare variances, Levene’s statistic was used. Neither ethnicity, BMI, nor menstrual phase was significantly related to body temperature nor did they interact in any significant way with sex, age, or time of day to affect temperature, so they were dropped from analyses. Sex, age, and time of day were included in all ANCOVA analyses.

It is likely that BMI was unrelated to any study outcomes because participants self-reported their height and weight, instead of our measuring that. For menstrual phase we could not analyze within-subject effects because temperatures were taken over only two-week periods. Between-subject analyses were hampered by failing to ask about the use of oral contraceptives, which presumably many women sampled were taking, and by less than half our female participants having ≥2 temperature readings during either their midluteal or early follicular menstrual phases.

**The first part of Hypothesis #1**, that there are sizeable individual differences in temperature, was confirmed. Inter-individual differences in oral temperature were large (see Table 1) and highly significant: F(93, 1237) = 18.99, p<0.001, partial eta squared (η_p_^2^) = 0.59. This was equally true for just temperature readings first thing in the morning (F[93, 570] = 19.26, p<0.001, η_p_^2^ = 0.76) or in the evening right before bed (F[93, 570] = 17.42, p<0.001, η_p_^2^ = 0.74). Note the exceptionally large effect sizes.

**Table 1:** Summary of oral temperature results in Celsius and Fahrenheit.

	N	Mean Age in years (sd)	% who were Female	**Mean Temp. (sd)**	Range of Mean Temp.	Range of Indiv. Temp. Readings	**Mean Temp. upon Waking (sd)**	Range of Mean Early a.m. Temp.	Range of Indiv. Temp. Readings in early a.m.
**All participants**	96	30.4 (10.7)	56%	**36.1°** (0.42)	35.2°–37.4°	32.4°–37.7°	**36.1°** (0.46)	35.0°–37.3°	33.5°–37.5°
				97.0° (0.076)	95.4°–99.3°				
**All Men**	42	30.6 (10.7)	n/a	**36.0°** (0.34)	35.4°–37.0°	32.4°–37.7°	**36.0°** (0.40)	35.0°–37.2°	33.5°–37.4°
**All Women**	54	30.2 (10.8)	n/a	**36.2°** (0.46)	35.2°–37.4°	33.8°–37.7°	**36.2°** (0.48)	35.3°–37.3°	34.4°–37.5°
**43–67 years olds**	19	46.6 (6.2)	58%	**36.0°** (0.20)	35.6°–36.4°	34.7°–37.2°	**36.0°** (0.28)	35.4°–36.5°	34.7°–37.2°
**18–42 years olds**	77	26.3 (7.1)	56%	**36.1°** (0.46)	35.2°–37.4°	32.4°–37.7°	**36.1°** (0.49)	35.0°–37.3°	33.5°–37.5°
**Older Women**	11	46.0 (4.9)	n/a	**36.0°** (0.14)	35.8°–36.2°	35.1°–36.9°	**36.1°** (0.27)	35.5°–36.5°	35.2°–36.9°
**Younger Women**	43	26.1 (7.7)	n/a	**36.3°** (0.50)	35.2°–37.4°	33.8 °- 37.7°	**36.3°** (0.52)	35.3°–37.3°	34.4°–37.5°
**Older Men**	8	47.5 (7.9)	n/a	**35.9°** (0.26)	35.6°–36.4°	34.7°–37.2°	**36.0°** (0.30)	35.4°–36.2°	34.7°–37.2°
**Younger Men**	34	26.6 (6.5)	n/a	**36.0°** (0.36)	35.4°–37.0°	32.4°–37.7°	**36.0°** (0.42)	35.0°–37.2°	33.5°–37.4°
	N	Mean Age in years (sd)	% who were Female	**Mean Temp. just before go- ing to bed** (sd)	Range of Mean Bedtime Temp.	Range of Indiv. Temp. Readings at bedtime	**Mean Change in Temp. overnight** (sd)	Range of Mean Over- night Change in Temp.	Range of Indiv. Temp. Change from Bedtime to Waking
**All participants**	96	30.4 (10.7)	56%	**36.1°** (0.45)	35.0°–37.3°	32.4°–37.7°	**-0.03°** (0.33)	-1.19° to 1.06°	-4.70° to 1.94°
				97.0° (0.81)	95.0°–99.1°	90.3°–99.9°	-0.05° (0.59)	-2.14° to 1.91°	-8.46° to 3.49°
**All Men**	42	30.6 (10.7)	n/a	**36.0°** (0.37)	35.1°–36.7°	32.4°–37.7°	**-0.03°** (0.38)	-1.19° to 1.06°	-4.70° to 1.94°
				96.8° (0.67)	95.2°–98.1°	90.3°–99.9°	-0.05° (0.68)	-2.14° to 1.91°	-8.46° to 3.49°
**All Women**	54	30.2 (10.8)	n/a	**36.2°** (0.48)	35.1°–37.4°	33.8°–37.7°	**-0.03°** (0.30)	-0.69° to 0.66°	-2.40° to 1.50°
				97.2° (0.86)	95.2°–99.3°	92.8°–99.9°	-0.05° (0.54)	-1.24° to 1.19°	-4.32° to 2.70°
**43–67 years olds**	19	46.6 (6.2)	58%	**36.0°** (0.27)	35.6°–36.7°	35.1°–37.1°	**-0.02°** (0.37)	-0.69° to 0.67°	-2.00° to 1.17°
				96.8° (0.49)	96.1°–98.1°	95.2°–98.8°	-0.04° (0.67)	-1.24° to 1.21°	-3.60° to 2.11°
**18–42 years olds**	77	26.3 (7.1)	56%	**36.1°** (0.48)	35.1°–37.4°	32.4°–37.7°	**-0.03°** (0.32)	-1.19° to 1.06	-4.70° to 1.94°
				97.0° (0.86)	95.2°–99.3°	90.3°–99.9°	-0.05° (0.58)	-2.14° to 1.91°	-8.46° to 3.49°
**Older Women**	11	46.0 (4.9)	n/a	**36.0°** (0.22)	35.6°–36.3°	35.1°–36.7°	**-0.03°** (0.39)	-0.69° to 0.61°	-1.30° to 1.11°
				96.8° (0.40)	96.1°–97.3°	95.2°–98.1°	-0.05° (0.70)	-1.24° to 1.10°	-2.34° to 2.00°
**Younger Women**	43	26.1 (7.7)	n/a	**36.2°** (0.52)	35.1°–37.4°	33.8°–37.7°	**-0.02°** (0.27)	-0.66° to 0.66°	-2.40° to 1.50°
				97.2° (0.94)	95.2°–99.3°	92.8°–99.9°	-0.04° (0.49)	-1.19° to 1.19°	-4.32° to 2.70°
**Older Men**	8	47.5 (7.9)	n/a	**36.0°** (0.33)	35.6°–36.7°	35.1°–37.1°	**0°** (0.36)	-0.40° to 0.67°	-2.00° to 1.17°
				96.8° (0.59)	96.1°–98.1°	95.2°–98.8°	32.0° (0.65)	-0.72° to 1.21°	-3.60° to 2.11°

Temperature values in the upper rows are in Celsius and those in the lower rows are in Fahrenheit.

Between-individual differences in temperature did not vary by gender, nor did variability just in the morning or evening temperatures. See Table 1.

Individual differences in temperature were larger among younger than older participants. Among participants 18–42 years old (mean = 26 years), range of mean temperatures spanned 2.2°C [3.9°F]. Among participants 43–67 years old (mean = 47 years), range of mean temperatures spanned 0.8°C [2.3°F]). The variance among younger adults was significantly greater than among those older (i.e., middle-aged): Levene statistic (1, 94) = 4.56, p<0.04. Similar age differences in temperature variability were seen in the morning and evening (Levene statistic (1,94) = 6.22, p<0.01; Levene statistic (1,94) = 5.94, p<0.02; respectively).

There was no difference in the size of individual differences in oral temperature by time of day. Variability among participants’ mean waking temperatures was comparable to that among their mean bedtime temperatures. The average morning time when participants took their temperature was 7:30; average evening time was 22:30.

**Fig 1:**
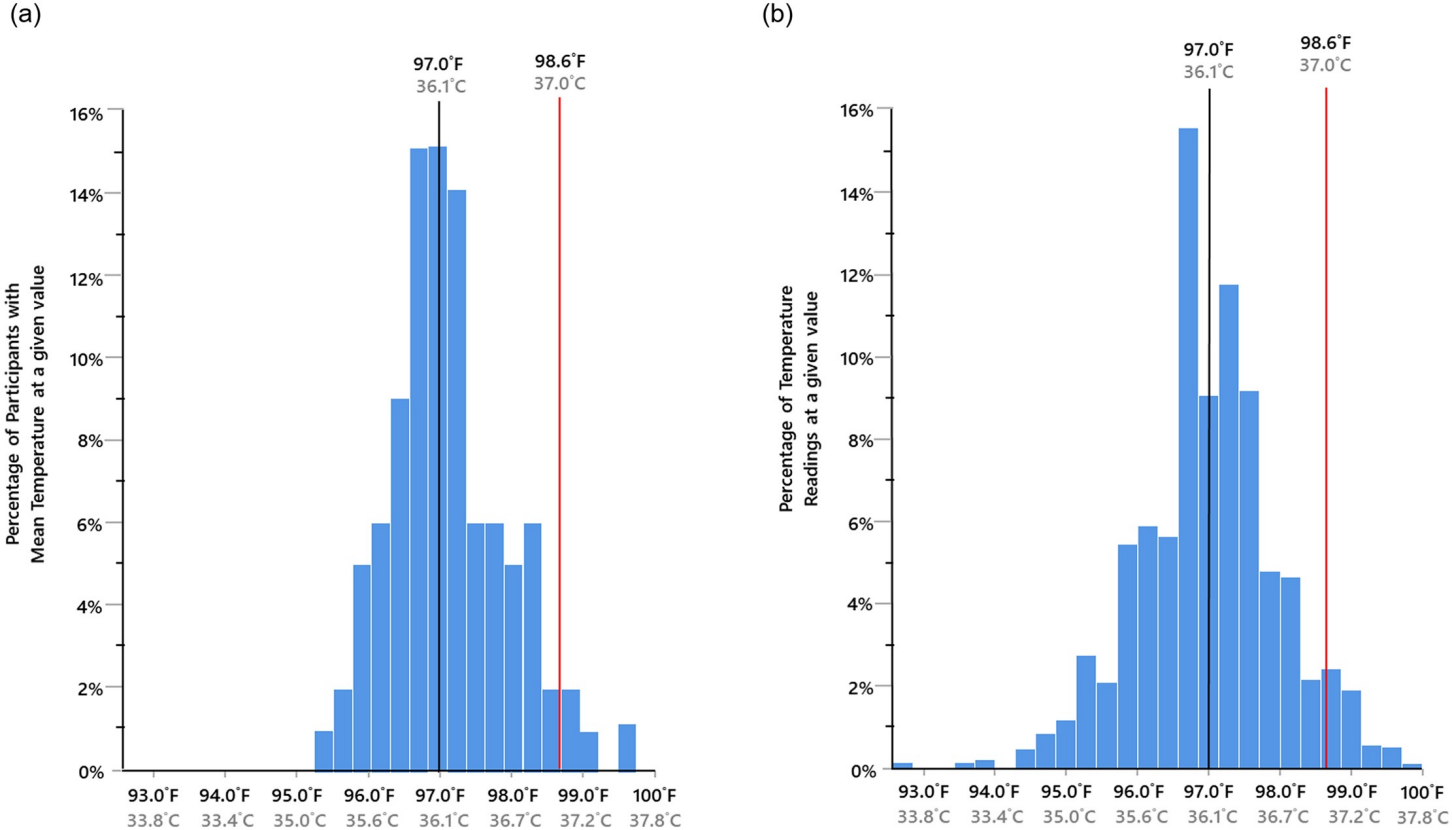
Distribution of the Mean Oral Temperatures of the 96 Study Participants (a). Distribution of all Oral Temperature Datapoints (N = 1,333)(b) 97.0°F indicates the mean body temperature across participants.

**The second half of Hypothesis #1**, that for many individuals their normal temperature is at least 1°F below 37.0°C (97.0°F), was also confirmed. Among our participants, 77% had a mean temperature ≤36.5°C (≤97.6°F). Significantly more participants had mean temperatures ≤36.5°Cithan above it: chi-square (1, N = 96) = 29.16, p<0.001. The mean temperature across all participants was 36.1°C (97.0°F), with a 95% confidence interval of 36.0°C (96.8°F) to 36.2°C (97.1°F). See Fig 1.

Among women participants, 82% had mean temperatures ≤36.5°C (97.6°F); the corresponding percentage for men was 71%. The difference between the percentage of men and women with mean temperatures ≤36.5°C (97.6°F) was not significant. Among adults ≥43 years old, 89% had mean temperatures ≤36.5°C (97.6°F); among adults 18–42 years that percentage was 77%. No adult ≥43 years had a mean temperature >36.4°C (97.5°F). Significantly more of our participants who had a mean temperature ≤36.5°C (97.6°F) were older than 42 versus younger: chi square (1, N = 74) = 21.62, p<0.001.

**Hypothesis #2**, that a given individual’s temperature is fairly stable, was confirmed too. The mean standard deviation across an individual’s 14 temperature readings was only 0.32°C (0.58°F). Across an individual’s seven morning readings it was 0.31°C (0.55°F) and across the seven evening readings it was 0.25°C (0.45°F). It was rare for a given individual’s temperature to vary by >0.55°C (1°F) over the 14 temperature readings at two timepoints per person; indeed only 7% of within-subject readings differed by that much. Variability among the 14 temperature readings per person did not vary by sex, age, or time of day.

Thermal variability also refers to diurnal variations in temperature. To look at that we paired morning and evening temperature measurements made on the same day in the same person. No significant differences emerged between a.m. and p.m. temperatures overall, or among either gender or age group.

**Hypothesis #3**, that women have higher body temperatures than women, was supported. Women participants tended to have higher body temperatures than the men (see Table 1). Though the difference was small, it was significant (F[1,91] = 9.59, p<0.003, η_p_^2^ = 0.09), but it accounted for only a tiny percentage of the variance and overlap in temperatures across men and women was substantial.

This sex difference was true for both morning (F[1,91] = 7.49, p<0.007, η_p_^2^ = 0.07) and higher morning temperatures than men (F[1,91] = 6.96, p<0.01, η_p_^2^ = 0.07). It was present among younger adults (F[1,74] = 7.28, p<0.01, η_p_^2^ = 0.09), but not among middle-aged ones. Indeed, among adults >42 years old, mean temperatures for men and women were identical.

We also examined this in just the 54 individuals in couples, where we have temperature readings from the wife and husband in the same room temperature wearing clothing of similar warmth. The mean temperature of the wives was 36.3°C (97.2°F); mean temperature of the husbands was 36.0 °C (96.7°F). Comparing the paired values controlling for age, this small difference (see Table 1) was significant: F(1,25) = 12.09, p<0.002, η_p_^2^ = 0.32. (We were surprised by such a large effect size for a mean difference of <1°, so we investigated further. The mean within-person standard deviation over the 14 readings was ~1% for husbands and for wives; the mean difference of the paired means had a 95% confidence interval of: 0.34° to 0.93°. The result of a paired t-test was t(25) = 4.40, p<0.001, R2 = 0.78.) Thus, even within couples, women had higher body temperatures than men. This sex difference within couples was true for both morning and evening temperature readings (morning: F[1,25] = 9.93, p<0.004, η_p_^2^ = 0.27; evening: F[1,25] = 18.70, p<0.001, η_p_^2^ = 0.42).

**Hypothesis #4**, that a person’s temperature tends to be higher in the evening than the morning, was disconfirmed. Indeed, the mean waking and bedtime temperatures of our participants were the same: 36.1°C (96.9°F). First-morning and bedtime temperatures did not vary by sex or age. Note that morning temperature readings were generally not taken during the window when lowest temperature is usually recorded (3–6 am); the mean morning time when temperatures were taken in our study was 7:30 am. Evening temperatures were generally taken by our participants at 10:30 pm (22:30), outside the window when peak temperature is normally recorded (4–9 pm [16:00–21:00]). Importantly, many of the temperature readings for our study might have been taken when participants were lying down. We return to these points in the discussion.

**Hypothesis #5**, that women are warmer in the evening and become cooler overnight but men’s temperatures show the opposite trend, received no support. To analyze this we looked at the difference in each pair of temperature readings for each participant (e.g., temperature on the night of X minus temperature on the morning of X+1). There was no sex or age difference in these difference scores. More than half our participants (57%) experienced an increase in temperature overnight. Overall, though, there was no consistent tendency for temperature to increase or decrease overnight.

Contrary to our prediction in **Hypothesis #6**, we found no age difference in average temperature overall, nor just in morning or evening. There was no indication whatever that younger adults tended to have higher or lower body temperatures than middle-aged adults.

We did not have a prediction concerning seasonal variation, but we compared temperatures taken before March (during the winter) with temperatures taken after March (during the Spring). We found no difference by season overall, nor among either gender or age group. In general, slightly warmer body temperature has been found in the summer, as in Lu et al.’s review [7] and several individual studies [10, 11], but at least one study found the opposite [12].

## Discussion

### Wide range of normal temperatures

Are there marked differences in the mean or “normal” temperature? Yes. The range of mean temperatures in our study spanned 2.2°C (4.0°F). Across a representative sample of reviews and other studies, the range in normal oral temperature has varied from 0.5°C (0.9°F) to 2.6°C (4.7°F; see Table 2). Other researchers who found variation in the normal temperature of healthy adults to be ≥2° include Geneva et al. [4], Sund-Levander et al. [13], Mackowiak et al. [5], Gomolin et al. [14, 15], and Keilson et al. [16] See Table 2 [17]. Individual temperature readings in our study ranged from 33.8°C (92.8°F) to 37.7°C (99.9°F); a difference of 3.9°C (7.1°F); see Fig 1b. Such large ranges mean that body temperature varies enough from person to person that using the same mean for everyone will cause marked errors.

**Table 2:** Results from a representative sample of studies and reviews of oral temperature in healthy adults.

Publications	# of Studies Reviewed that assessed oral temp.	Number of subjects	Mean Age in years (sd)	Age Range in years	% who were Female	Where partici- pants lived	Mean Temp. (sd)	Range of Mean Temp.	Size of the Range	Mean time of the a.m. temp. reading	Mean a.m. Temp. (sd)	Range of Early a.m. Temp.	Mean time of the p.m. temp. reading	Mean p.m. Temp. (sd)	Range of p.m. Temp.
**Present study**		96	30.4 (10.7)	18–67	56%	com- munity	**36.1°** (0.42)	35.2°–37.4°	2.2°	7:30	**36.1°** (0.46)	35.0°–37.3°	22:30	**36.1°** (0.45)	35.0°–37.3°
Geneva et al. (2019)*	15		all <60 yrs		n/a	com- munity	**36.7°** (0.34)	35.7°–37.4°	1.7°						
Geneva et al. (2019)*	19		all ≥60 yrs		n/a	n/a	**36.4°** (0.48)								
**Waalen & Buxbaum (2011)**		9,227	57.3 (13.5)	20–98	100%	n/a	**36.4°** (0.66)								
**Waalen & Buxbaum (2011)**		9,403	58.6 (13.3)	20–98	0%	n/a	**36.2°** (0.60)								
**Lu et al. (2009)**	16		all ≥60 yrs		n/a	n/a	**36.3°** (0.09)	36.1°–36.6°	0.5°		**36.0°** (0.10)			**36.4°** (0.09)	
**Gomolin et al. (2005)**		100	65–98			nursing home				6:00	**36.3°**	34.4°–37.1°	22:00	**36.6°**	35.6°–37.6°
**Gomolin et al. (2005)**		50	81	65–98		com- munity	**36.5°**	35.4°–37.3°	1.9°						
**Gomolin et al. (2007)**		167	82.5 (8.8)		70%	nursing home				7:00	**36.3°** (0.46)	34.4°–37.2°	16:30	**36.3°** (0.51	35.1°–37.6°
**Mackowiack et al. (1992)**		148	18–40		18%	com- munity	**36.8°** (0.4°)	35.6°–38.2°	2.6°						
**Mason et al (1988)**		18	72	65–80	100%	com- munity	**36.4°**	35.9°–36.8°	0.9°						
**Keilson et al. (1985)**		20	32	22–43	45%	com- munity				early am; 1st awak- ening	**36.4°** (0.42)				
**Keilson et al. (1985)**		97	74	65–90	68%	com- munity					**36.2°** (0.42)	35.4°–37.0°			
**Primrose and Smith (1982)**		220	60–94				**36.0°**	35.5°–37.3°	1.8°						
**Wunderlich (1868)**		25,000				hospital	**37.0°**	36.2°–37.5°	1.3°						

### Marked stability of temperature over 14 readings spanning 2 weeks

The stability of temperature readings taken at roughly the same times across several days had not previously been reported. We found it to be quite stable. Thermal stability was equally true for men and women, and older (i.e., middle-aged) and younger adults. This is consistent with a snapshot of someone’s temperature taken at each yearly check-up being sufficient to establish the average temperature for that person, realizing that slight dips very early morning and slight increases just before and after dusk are typical [2, 5, 7–9, 14].

We did not find the diurnal changes that are so well established. That was probably because of the times of day when we assessed temperature (07:00, after the morning nadir; and 22:30, after the peak around dusk; [5, 8, 18]). While studies find that temperatures tend to rise in late afternoon, they also find they dip just before sleep onset [19–21]. Also, many of the temperature measurements for our study may have been taken while participants were in bed (after having just awakened or before going to sleep). Prone or recumbent posture has associated with lower temperature [20, 22, 23]. This may also be why the temperatures we observed were toward the lower end of the range of temperatures found in other studies. Also our study is more recent and Protsiv et al. [24] report that temperatures have been declining.

### 37.0°C (98.6°F) is not an accurate approximation of the normal temperature of most adults

The mean oral temperature of our healthy volunteers was 36.1°C (97.0°F). The mean temperatures from reviews and studies listed in Table 2 vary from 36.0°C (96.8°F) to 36.8 °C (98.2°F), except for Wunderlich. Wunderlich’s estimate is especially too high when oral versus rectal thermometers are used because oral and axillary temperatures tend to be 0.3–0.6°C (0.5–1.0°F) lower than rectal temperature, though the range is similar [7, 13, 25]. Experts have been arguing to abandon 37.0°C (98.6°F) as not having any “special significance vis-à-vis the oral temperature of healthy adults” since at least 1992 [5 p. 1580]. A just-published study based on massive datasets [24] reports that at least in the U.S., temperatures have been declining at the rate of 0.03°C per decade of birth.

The mean currently in use (~37.0°C [~98.6°F]) is too high, but this has persisted despite 35 years of research consistently showing that it’s too high. Using this mean causes fevers in those with normally lower temperatures to be missed.

Medical historians trace the wide acceptance that normal body temperature of healthy adults is ~37.0°C (98.6°F) primarily to Wunderlich [1]. Wunderlich was the first to systematically use a thermometer to measure human body temperature. His data are based on ~25,000 patients, whose care he supervised. There are a number of limitations with this classic work, however.^a^

Whereas most researchers and clinicians before us have argued for a new, lower estimate of normal adult temperature, we are urging that normal temperature be personalized because what is normal for one person can be quite different from what is normal for another. As Edelsberg [27] wrote: “[N]o single value of mean body temperature has much clinical importance because of individual and population variability.”

We are particularly worried about fevers not being detected in persons with low normal temperatures (e.g., ≤36.5°C [97.6°F]), which in our study was 77% of participants, 82% of just the women, and 89% of adults ≥43 years age. Indeed, 30% of our sample had mean temperatures <35.9°C (96.6°F). Similarly, Waalen et al. [3 pp. 490–491] wrote: “[I]n every age cohort up to age 50, there is a small discrete subset of individuals with body temperatures less than 96°F. The representation of that population increases with increasing age.” Since studies consistently report lower mean temperatures in seniors than in younger adults, it is particularly likely that troubling temperature elevations in elderly adults might be missed if one “normal” temperature continues to be assumed for everyone at all ages.

Thus, all of our principal hypotheses were confirmed. We turn now to our secondary hypotheses; only one of which were confirmed.

### Our secondary hypotheses

More than half our participants (57%) experienced a temperature rise overnight, but overall there was no consistent trend in either direction. Others have shown that temperature is lower during sleep and its onset [22, 28, 29]. Two studies had previously examined overnight temperature fluctuations, though both looked at only one night per participant, did not align temperature changes to when a participant actually fell asleep or awoke, and had small samples (Baker et al. [21]; Cagnacci et al. [30]).^a^^b^

We found that women had slightly higher temperatures than men, consistent with Wunderlich et al. [6], Mackowiak et al. [5], and Sund-Levander et al. [13], but the opposite of that reported in McGann et al.’s study [31] or Geneva et al.’s review [4]. As far as we know, no one had previously compared the temperatures of spouses. We asked couples to be in the same room before and during taking their temperatures and to wear clothing of similar warmth. Even within couples we found that women had slightly higher temperatures than men. However, if there is a reliable sex difference in adults’ temperatures it seems tiny and unlikely to be of clinical significance.

Women tend to have higher temperatures in the midluteal phase [2, 21, 30]. Our sex-difference results do not seem to be menstrual-phase driven, however, since only 12 women measured their temperature even once during their estimated midluteal phase. We found a trend for temperatures to be higher during the midluteal phase, but that was not significant. Another reason we might have found higher temperatures in women than men is that many of our female participants were probably taking oral contraceptives. Women’s temperatures tend to be higher when they are on oral contraceptives [21, 32].

Although it has been consistently reported that older adults have lower temperatures than younger adults [2–6, 9], we did not find that. Almost certainly that is because our older adults were middle-aged rather than elderly. The mean age of our older adults was 47 years and only two participants were ≥60 years. The participants in Mackowiak et al. [5] were 18–40 and no age-related difference in temperature was found. The participants in Adhi et al. [33] were 9–70 (mean age = 34 years) and they, too, found no age-related temperature difference.

We did find a larger range of temperatures among younger adults, but no greater diurnal variability. Perhaps we found no age difference in diurnal temperature fluctuations because of when we assessed temperatures or because our participants were more similar than different in their degree of physical activity. Thermal variability is greater in those more physically active [34].

### Limitations

Limitations of our study include a small sample size and our not having asked about use of oral contraceptives or level of physical activity. We should have asked participants to note the room temperature when taking each temperature reading and note their posture. We had only two participants ≥60 years old, which limited our ability to look at age differences throughout the adult lifespan. We did not confirm menstrual phases with hormone assays. Not having assessed temperatures between 16:00–21:00, we didn’t include the window of peak temperatures. We should have measured participants’ height and weight ourselves rather than accepting participants’ self-reports. It might have been more accurate, though less practical, if we had taken the temperature readings ourselves, but given the small variability between readings and our means and ranges being comparable to those found by others, participants seem to have accurately measured their temperatures.

### Conclusions

Our take-home message is that it is time to personalize body temperature. It is needed and easily doable. The range in normal temperature is sufficiently large that using a standard “normal” temperature value will lead to errors for many individuals. For the many people whose normal temperature is much lower than 37.0°C (98.6°F), healthcare professionals may fail to identify a pathologically high fever or may obtain false negatives when using oral temperature to screen for COVID-19, mistaking what is actually an elevated temperature for normal.

We propose that a person’s temperature be taken at each doctor visit, just as we do now with blood pressure. This would be simple to implement and would permit an accurate estimate of each person’s normal temperature because, as we have shown, temperature varies little across days. Such records over time would also provide a more accurate understanding of how temperature changes over the lifespan.

## Footnotes

^a^ It was conducted with patients (i.e., nonhealthy individuals). Temperature readings were axillary and the thermometer had to be held in place for 4–8 minutes. Those old mercury-in-glass thermometers had to be read in situ. His thermometers were not as precise as today’s and were probably calibrated differently, yielding readings ~1.7°C (3.1°F) higher than those obtained with digital thermometers [2]. Medical historians question whether >1,000,000 temperature readings were really taken when the thermometers took 15–20 min to calibrate, and even if taken, whether with the technology available Wunderlich could have analyzed more than a fraction of those data [26].

^b^Baker et al.’s Fig 1 indicates that temperature dropped precipitously in men right after sleep onset, not fully regaining its bedtime level by waking. Temperature declined less in women during sleep, and by waking regained almost its entire sleep-related loss. Women taking contraceptive medication had the highest bedtime temperatures; by waking their temperature had risen only to the same level as that for ovulating women. Cagnacci et al. found comparable results to Baker et al. for normally-cycling women; they didn’t include men or women on oral birth control.
