# Correction: One Size Does Not Fit All: Assuming the Same Normal Body Temperature for Everyone is Not Justified

**DOI:** 10.1371/journal.pone.0259428

**Published:** 2021-10-29

**Authors:** Adele Diamond, Carolyn T. Lye, Deepali Prasad, David Abbott

In Table 1, the last row “Younger Men” is missing. There are also errors in how the temperature ranges are displayed throughout the table and multiple cells in the fourth column are incorrectly written as “n/a”. Please see the correct table here.

**Table 1:** Summary of oral temperature results in Celsius and Fahrenheit.

	N	Mean Age in years (sd)	% who were Female	**Mean Temp.** (sd)	Range of Mean Temp.	Range of Indiv. Temp. Readings	**Mean** **Temp.** **upon Waking** (sd)	Range of Mean Early a.m. Temp.	Range of Indiv. Temp. Readings in early a.m.
**All participants**	96	30.4 (10.7)	56%	**36.1°** (0.42) 97.0˚ (0.76)	35.2° - 37.4° 95.4^°^ - 99.3^°^	32.4° - 37.7° 90.3° - 99.9°	**36.1°** (0.46) 97.0° (0.83)	35.0° - 37.3° 95.0° - 99.1°	33.5° - 37.5° 92.3° — 99.5°
**All Men**	42	30.6 (10.7)	0	**36.0°** (0.34) 96.8˚ (0.61)	35.4° - 37.0° 95.7˚ - 98.6˚	32.4° - 37.7° 90.3° - 99.9°	**36.0°** (0.40) 96.8° (0.72)	35.0° - 37.2° 95.0° - 99.0°	33.5° - 37.4° 92.3° - 99.3°
**All Women**	54	30.2 (10.8)	100%	**36.2°** (0.46) 97.2˚ (0.83)	35.2° - 37.4° 95.4˚ - 99.3˚	33.8° - 37.7° 92.8° - 99.9°	**36.2°** (0.48) 97.2° (0.86)	35.3° - 37.3° 95.5° - 99.1°	34.4° - 37.5° 93.9° - 99.5°
**43–67 years olds**	19	46.6 (6.2)	58%	**36.0°** (0.20) 96.8˚ (0.36)	35.6° - 36.4° 96.1˚ - 97.5˚	34.7° - 37.2° 94.5° - 99.0°	**36.0°** (0.28) 96.8° (0.50)	35.4° - 36.5° 95.7° - 97.7°	34.7° - 37.2° 94.5° - 99.0°
**18–42 years olds**	77	26.3 (7.1)	56%	**36.1°** (0.46) 97.0˚ (0.83)	35.2° - 37.4° 95.4˚ - 99.3˚	32.4° - 37.7° 90.3° - 99.9°	**36.1°** (0.49) 97.0° (0.88)	35.0° - 37.3° 95.0° - 99.1°	33.5° - 37.5° 92.3° - 99.5°
**Older Women**	11	46.0 (4.9)	100%	**36.0°** (0.14) 96.8˚ (0.25)	35.8° - 36.2° 96.4˚ - 97.2˚	35.1° - 36.9° 95.2° - 98.4°	**36.1°** (0.27) 97.7° (0.49)	35.5° - 36.5° 95.9° - 97.7°	35.2° - 36.9° 95.4° - 98.4°
**Younger Women**	43	26.1 (7.7)	100%	**36.3°** (0.50) 97.3˚ (0.90)	35.2° - 37.4° 95.4˚ - 99.3˚	33.8 °- 37.7° 92.8° - 99.9°	**36.3°** (0.52) 97.3° (0.94)	35.3° - 37.3° 95.5° - 99.1°	34.4° - 37.5° 93.9° - 99.5°
**Older Men**	8	47.5 (7.9)	0	**35.9°** (0.26) 96.6˚ (0.47)	35.6° - 36.4° 96.1˚ - 97.5˚	34.7° - 37.2° 94.5° - 99.0°	**36.0°** (0.30) 96.8° (0.54)	35.4° - 36.2° 95.7° - 97.2°	34.7° - 37.2° 94.5° - 99.0°
**Younger Men**	34	26.6 (6.5)	0	**36.0°** (0.36) 96.8˚ (0.65)	35.4° - 37.0° 95.7˚ - 98.6˚	32.4° - 37.7° 90.2° - 99.0°	**36.0°** (0.42) 96.8° (0.76)	35.0° - 37.2° 95.0° - 99.0°	33.5° - 37.4° 92.3° - 99.3°
	N	Mean Age in years (sd)	% who were Female	**Mean** **Temp. just before going to bed** (sd)	Range of Mean Bedtime Temp.	Range of Indiv. Temp. Readings at bedtime	**Mean** **Change in Temp. overnight** (sd)	Range of Mean Overnight Change in Temp.	Range of Indiv. Temp. Change from Bedtime to Waking
**All participants**	96	30.4 (10.7)	56%	**36.1°** (0.45) 97.0˚ (0.81)	35.0° - 37.3° 95.0˚ - 99.1˚	32.4° - 37.7° 90.3° - 99.9°	**-0.03°** (0.33) -0.05° (0.59)	-1.19° to 1.06° -2.14° to 1.91°	-4.70° to 1.94° -8.46° to 3.49°
**All Men**	42	30.6 (10.7)	0	**36.0°** (0.37) 96.8˚ (0.67)	35.1° - 36.7° 95.2˚ - 98.1˚	32.4° - 37.7° 90.3° - 99.9°	**-0.03°** (0.38) -0.05° (0.68)	-1.19° to 1.06° -2.14° to 1.91°	-4.70° to 1.94° -8.46° to 3.49°
**All Women**	54	30.2 (10.8)	100%	**36.2°** (0.48) 97.2˚ (0.86)	35.1° - 37.4° 95.2˚ - 99.3˚	33.8° - 37.7° 92.8° - 99.9°	**-0.03°** (0.30) -0.05° (0.54)	-0.69° to 0.66° -1.24° to 1.19°	-2.40° to 1.50° -4.32° to 2.70°
**43–67 years olds**	19	46.6 (6.2)	58%	**36.0°** (0.27) 96.8˚ (0.49)	35.6° - 36.7° 96.1˚ - 98.1˚	35.1° - 37.1° 95.2° - 98.8°	**-0.02°** (0.37) -0.04° (0.67)	-0.69° to 0.67° -1.24° to 1.21°	-2.00° to 1.17° -3.60° to 2.11°
**18–42 years olds**	77	26.3 (7.1)	56%	**36.1°** (0.48) 97.0˚ (0.86)	35.1° - 37.4° 95.2˚ - 99.3˚	32.4° - 37.7° 90.3° - 99.9°	**-0.03°** (0.32) -0.05° (0.58)	-1.19° to 1.06 -2.14° to 1.91°	-4.70° to 1.94° -8.46° to 3.49°
**Older Women**	11	46.0 (4.9)	100%	**36.0°** (0.22) 96.8˚ (0.40)	35.6° - 36.3° 96.1˚ - 97.3˚	35.1° - 36.7° 95.2° - 98.1°	**-0.03°** (0.39) -0.05° (0.70)	-0.69° to 0.61° -1.24° to 1.10°	-1.30° to 1.11° -2.34° to 2.00°
**Younger Women**	43	26.1 (7.7)	100%	**36.2°** (0.52) 97.2˚ (0.94)	35.1° - 37.4° 95.2˚ - 99.3˚	33.8° - 37.7° 92.8° - 99.9°	**-0.02°** (0.27) -0.04° (0.49)	-0.66° to 0.66° -1.19° to 1.19°	-2.40° to 1.50° -4.32° to 2.70°
**Older Men**	8	47.5 (7.9)	0	**36.0°** (0.33) 96.8˚ (0.59)	35.6° - 36.7° 96.1˚ - 98.1˚	35.1° - 37.1° 95.2° - 98.8°	**0°** (0.36) 32.0° (0.65)	-0.40° to 0.67° -0.72° to 1.21°	-2.00° to 1.17° -3.60° to 2.11°
**Younger Men**	34	26.6 (6.5)	0	**36.0**° (0.39°) 96.8° (0.70°)	35.1° to 36.7° 95.2° to 98.1°	32.4° to 37.7° 90.3° to 99.9°	**-0.03**° (0.39°) -0.05° (0.70°)	-1.19° to 1.06° -2.1° to 1.9°	-4.70° to 1.94° -8.5° to 3.5°

Temperatures in the upper rows are in Celsius and those in the lower rows are in Fahrenheit.
